# Prevalence of strabismus among school going children in India

**DOI:** 10.6026/973206300210410

**Published:** 2025-03-31

**Authors:** Saniana Anil Hegde, Joshi B.S, Karambelkar V.H

**Affiliations:** 1 Department of Ophthalmology, Krishna Institute of Medical Sciences, Krishna Vishwa Vidyapeeth (Deemed to Be University), Karad, Maharashtra, India

**Keywords:** Strabismus, rural, urban, monitor, prevalence

## Abstract

Children with strabismus (crossed eyes" or "squint) may have functional issues with reading and other academic activities, possibly resulting in reduction of overall academic achievement. Therefore, it is of interest to evaluate the prevalence of strabismus and its role with total of 995 students randomly divided between rural and urban area during camp with their relative histories, screen time, outdoor activity, reading time and ocular examination. We found difference for all the variables with no statistical significance. Hence, it is essential to monitor these children closely and intervene before their situation deteriorates to prevent this condition from adversely affecting their vision or academic performance.

## Background:

In strabismus, the ocular alignment is disrupted, leading to the patient perceiving their environment through misaligned visual axes. Strabismus and esotropia are terms commonly used to describe this condition [1]. Early identification and treatment are crucial in preventing long-term visual and psychological complications [2]. Strabismus is a significant public health concern, particularly among children who attend school regularly [3]. In assessing the morbidity associated with ST, it is crucial to examine the impact of the condition on a child's visual acuity, academic achievement and overall quality of life [4]. The morbidity found linked to Strabismus was seen associated with a range of functional, psychological and visual outcomes. Amblyopia, often referred to as "lazy eye," is a condition where one eye exhibits diminished visual acuity compared to the other. This occurs due to the brain's preferential treatment of one eye, leading to the underdevelopment of the visual pathways associated with the affected eye [5]. Children diagnosed with strabismus may experience functional challenges in reading and other educational activities, which could result in a decline in academic performance [6]. Individuals experiencing attention difficulties, diplopia, or ocular fatigue may encounter considerable obstacles when participating in extended visual activities. This can impact a child's sense of self and social relationships, affecting both psychological and social dimensions [7]. An individual's visibly mismatched eyes can lead to social isolation and significant psychological distress when bullied or ridiculed. This may also affect the child's self-assurance and receptiveness [8, 9]. Therefore, it is of interest to report the prevalence of strabismus among Indian school going children.

## Materials and Methods:

The current retrospective cross sectional observational study was conducted in school going Karad Taluka with 955 samples in total. Students who were identified with Strabismus during camp screenings were subsequently monitored in the out-patient department with their history (birth, developmental, family, past, spectacle use, ocular trauma, surgery history, past infection, screen exposure time (mobile and TV use), hours of outdoor activity and constant reading time). To perform ocular examination we have evaluated visual acuity recording, visual axis assessment, ocular movement assessment and cycloplegic refraction with fundoscopy.

## Inclusion criteria:

[1] School going children

[2] 5 to 14 years

## Exclusion criteria:

Children below 5 year and above 14 years

## Statistical analysis:

Using the Chi-square and Fisher exact tests, data were analyzed. It was considered statistically significant if the p-value was less than 0.

## Results:

In this study 52.8% were male students and 47.2% were female students (Table 1). Table 2 shows the high prevalence of strabismus in females (1.2%) than males (0.9%), but the relation is not statistically significant (p=0.7644). Table 3 shows that out of the total of 1051 students 30.4 % belong to age group of 5-8 years, 35.4% belong to age group of 9-11 years, 34.2% belong to age group of 12-14 years. Table 4 shows that 5-8 years old is 0.6%, 9-11 years is 0.5% and in 12-14 years is 1.9%. Table 5 shows that, 67.4% belonged to rural 32.6 % percentage belonged to urban region. Table 6 shows the prevalence in rural 6 (0.8%) than in urban 5 (1.5%) respectively. Table 7 shows that, almost same strabismus with or without with 50% students. Table 8 shows that majority showed myopia with 41 patients (4%) followed by hyper-metropia and astigmatism with 11 patients (1%) respectively. Majority of the students were showing without strabismus for both myopia and Hypermetropia on comparison with Strabismus respectively (Table 9). Table 10 shows that, majority of the students showed exotropia with 6 patients (75%) for myopia on the other hand, esotropia had 2 patients (25%) respectively. For Hypermetropia, esotropia showed majority 3 patients (100%) respectively. Table 11 shows that the number of students for prevalence was seen in 11 patients (1.04%) respectively. Table 12 shows that, majority of the students had reading time with 3 patients (30-60 min) in urban area for esotropia while 2 patients (30-60min) for exotropia respectively. At rural area, 2 patients (30-60 min) esotropia while 3 patients (<30 min) and 1 patients (30- 0min) for exotropia respectively. Thus, it showed non-significant difference. Table 13 shows that, majority of the students had Mobile use with 2 patients (30-60 min) in urban area for esotropia and <30min for 1 patients for esotropia and 2 patients (>60min) for exotropia, respectively. At rural area, 2 patients (<30min) esotropia while 3 patients (<30 min) and 1 patients (30-60min) for exotropia respectively and it showed non-significant difference. Table 14 shows that, majority of the students had TV with 2 patients (30-60 min) in urban area for esotropia 1 patients (<30min). While, on the other hand, 1 patients (30-60 min and >60min) for exotropia respectively. At rural area, 1 patients (30-60min and >60min) esotropia while 2 patients (<30 min) and 1 patients (30-60min and >60 min) for exotropia respectively and it is not a significant difference. Table 15 shows that, majority of the students had OA with 2 patients (>60min) and 1 (<30 min) in urban area for exotropia 2 patients (<30min). At rural area, 1 patients (30-60min and >60min) esotropia while 2 patients (30-60 min) and 1 patients (<30 min) for exotropia respectively and it is not a significant difference.

## Discussion:

A total of 1051 students were included with the mean age of the patients was 10.07±2.88 in this study. No instances of paralytic squint or amblyopia were reported. In this population, exotropia is more prevalent than estropia, with an estimated Strabismus prevalence of 1.04%. 52.8% of the participants in this study were male students, while 47.2% were female students. Females had a frequency of 1.2%, which lacks statistical significance. The prevalence of Strabismus was greater in the urban population (1.5%); however, this association is statistically insignificant. Strabismus manifested in 50% of students with a familial predisposition to the illness. The relationship is statistically significant. As shown in Table 16, Table 17 and Table 18 by comparing various different studies results with our study results. The prevalence of strabismus was 31 (5.0%); 95% confidence interval: 3.45, 6.97. A family history of strabismus (AOR= 3.9 (95% CI: 1.71-11.22)), hyperopia of - +3.00 diopters sphere (AOR=5.3 (95% CI: 2.01, 10.77)) and not breastfeeding exclusively (AOR= 2.9 (95% CI: 1.14-4.71)) were the only risk factors for strabismus. Thus, they come to conclude that, the prevalence of strabismus among youngsters residing in Bahr Dar city was around 5% [19] in another study, the prevalence of strabismus in Lhasa Childhood Eye Study was 3.7%, which was higher than previous reports from Chinese childhood epidemiology studies. Strabismus is a common contributing factor to amblyopia [20].

## Conclusion:

Strabismus is common among school going children. Thus, it is important to keep an eye on these kids and take action before they get worse, so that this condition doesn't affect their eyesight or their ability to learn. Future research should explore the influence of environmental factors and genetic predispositions on the prevalence of Strabismus across various populations.

## Figures and Tables

**Table 1:** Gender distribution

**Gender**	**Frequency**	**Percentage**
Males	554	52.80%
Females	497	47.20%

**Table 2:** Gender strabismus

**Gender**	**With strabismus**	**Without strabismus**	**Total**
Male	5(0.9%)	549(99.1%)	554(100%)
Female	6(1.2%)	491(98.8%)	497(100%)
Total	11(1.04%)	1040(98.96%)	1051(100%)

**Table 3:** Age distribution

**Age group**	**Total number of students**	**Percentage**
8-May	320	30.40%
11-Sep	372	35.40%
14-Dec	359	34.20%
Total	1051	100

**Table 4:** Strabismus (age)

**Age group**	**With strabismus**	**Without strabismus**	**Total**
8-May	2(0.6%)	318(99.4%)	320(100%)
11-Sep	2(0.5%)	370(99.5%)	372(100%)
14-Dec	7(1.9%)	352(98.1%)	359(100%)
Total	11(1.04%)	1040(98.96%)	1051(100%)

**Table 5:** Region distribution

**Region**	**Total No. of Students**	**Percentage**
Urban	343	32.60%
Rural	708	67.40%
Total	1051	100%

**Table 6:** Strabismus with region

**Region**	**With strabismus**	**Without strabismus**	**Total**
Urban	5(1.5%)	338(98.5%)	343(100%)
Rural	6(0.8%)	702(99.2%)	708(100%)
Total	11(1.04%)	1040(98.96%)	1051(100%)

**Table 7:** Family history

**Family H/O**	**With strabismus**	**Without strabismus**	**Total**
Present	3(50%)	3(50%)	6(100%)
Absent	8(0.8%)	1037(99.2%)	1045(100%)
Total	11(1.04%)	1040(98.96%)	1051(100%)

**Table 8:** Refractive error

**Type of refractive error (refractive error)**	**Total no. of students**	**Percentage (1051)**
Myopia (MP)	41	4%
Hypermetropia (HMR)	11	1%
Astigmatism (AGM)	11	1%
Total	63	6%

**Table 9:** Strabismus with refractive error

**Refractive error**	**With Strabismus**	**Without strabismus**	**Total**
Myopia	8(19.5%)	33(80.5%)	41(100%)
Hypermetropia	3(27.3%)	8(72.8%)	11(100%)

**Table 10:** Types of Strabismus

**Type of refractive error**	**Esotropia (et)**	**Exotropia (ex)**	**Total**
Myopia	2(25%)	6(75%)	8(100%)
Hypermetropia	3(100%)	0	3(100%)
Astigmatism	0	0	0
Total	5	6	11(100%)

**Table 11:** Prevalence

**No. of students with strabismus**	**Total number of participants**
11(1.04%)	1051(100%)	

**Table 12:** Reading time

**Region**		**Constant reading time**			**Total**
		**<30minutes**	**30-60 minutes**	**>60 minutes**	
Urban	Esotropia	0	3	0	3
	Exotropia	0	2	0	2
Rural	Esotropia	0	2	0	2
	Exotropia	3	1	0	4
Total		3	8	0	11

**Table 13:** Mobile use

**Region**		**Mobile usage time**			**Total**
		**<30minutes**	**30-60 minutes**	**>60 minutes**	
Urban	Esotropia	1	2	0	3
	Exotropia	0	0	2	2
Rural	Esotropia	2	0	0	2
	Exotropia	3	1	0	4
Total		4	5	2	11

**Table 14:** TV

**Region**		**TV Usage hours**			**Total**
		**<30minutes**	**30-60 minutes**	**>60 minutes**	
Urban	Esotropia	1	2	0	3
	Exotropia	0	1	1	2
Rural	Esotropia	0	1	1	2
	Exotropia	2	1	1	4
Total		4	5	2	11

**Table 15:** Outdoor activity (OA)

**Region**		**Outdoor activity**			**TOTAL**
		**<30minutes**	**30-60 minutes**	**>60 minutes**	
Urban	Esotropia	1	0	2	3
	Exotropia	2	0	0	2
Rural	Esotropia	0	1	1	2
	Exotropia	1	2	1	4
Total		4	5	2	11

**Table 16:** Prevalence

**Author**	**Year**	**Sample**	**Region**	**Age (years)**	**Prevalence**
Current	2021-2024	1052	Karad	14-Mar	1.04%
Graham *et al.* [10]	1974	4784	Cardiff, England	6-May	7.10%
Pratap *et al.* [11]	1989	3490	North India	-	2.87% primary 0.4%paralytic
Gupta *et al.* [12]	2000	1561	-	16-Jun	2.50%
Attada *et al.* [8]	2016	50	Vishakhapatnam	16-Mar	0.60%
Singh *et al.* [13]	2017	4838	West Uttar Pradesh	15-May	0.27%
Mittal *et al.* [14]	2022	13492	Uttarakhand	16-Jun	0.60%
Satav *et al.* [15]	-	4357	Melghat	18-Jun	0.41%

**Table 17:** Esotropia, Exotropia and age distribution

**Author**	**Year**	**Study sample**	**Region**	**Age(Year)**	**Esotropia**	**Exotropia**
Kothari *et al.* [16]	2009	93 prevalence	Maharashtra	16-Apr	44%	56%
Agarwal *et al.* [17]	2016	1557	Chhattisgarh	15-May	0	100%

**Table 18:** Age and prevalence

**Author**	**Year**	**Study sample**	**Region**	**Age**	**Prevalence**	**Boys**	**Girls**
Graham *et al.* [18]	1974	4784	Cardiff, England	6-May	7.10%	7.30%	6.90%
Mittal *et al.* [14]	2022	13492	Uttarakhand	16-Jun	0.60%	More	-
Attada *et al.* [8]	2012-2014	50	Visakhapatnam	16-Mar	0.60%	50.85%	49.15%

## References

[1] Zhang X.J (2021). Scientific Reports..

[2] Agaje B.G (2020). Journal of International Medical Research..

[3] Bommireddy T (2023). Paediatrics and Child Health..

[4] Tegegne M.M (2021). Clinical Optometry..

[5] Hashemi H (2019). Strabismus..

[6] Dirani M (2010). Investigative ophthalmology and visual science..

[7] Wang Y (2021). BMJ open..

[8] Attada T.R (2016). Int J Res Med Sci..

[9] Kothari M (2009). Indian journal of ophthalmology..

[10] Graham P.A (1974). The British journal of ophthalmology..

[11] Pratap V.B, Lal H.B (1989). Indian Journal of Ophthalmology..

[12] Gupta M, Gupta Y (2000). Indian J Public Health..

[13] Singh A (2017). Indian journal of ophthalmology..

[14] Mittal S.K (2022). Indian Journal of Ophthalmology..

[15] Satav K.A (2023). Indian Journal of Medical Research..

[16] Kothari M (2009). Indian journal of ophthalmology..

[17] Agarwal A.B (2016). Investigative ophthalmology and visual science..

[18] Graham P.A (1974). The British journal of ophthalmology..

[19] Tegegne M.M (2021). Clinical Optometry..

[20] He H (2020). BMC ophthalmology..

